# Tobacco-Alcohol Toxic Optic Neuropathy

**Published:** 2019

**Authors:** Farah Sunnar Constantin, Monica Irina Ion, Andrei Erol Constantin

**Affiliations:** * Department of Ophthalmology, Clinical Emergency County Hospital, Constanţa, Romania; ** Faculty of Medicine, “Ovidius” University Constanţa, Romania; *** ”Carol Davila” University of Medicine and Pharmacy Bucharest, Romania

**Keywords:** toxic optic neuropathies, decrease of vision, bilateral

## Abstract

**Purpose.** To present the case of a 34-year-old man with sudden bilateral decrease of visual acuity, followed by a complete and fast recovery.

**Material and methods.** We reported a case of an apparently healthy 34-year-old patient with sudden and unpainful decrease of visual acuity, presumed of toxic etiology. The patient was treated at the debut of the symptomatology, thus having a favorable evolution during hospitalization, with the full recovery of his visual acuity, without complications.

**Conclusions.** Tobacco-Alcohol Toxic Optic Neuropathy has an unknown pathophysiological mechanism that allows very good recovery of visual acuity, if treated early.

## Introduction

Toxic Optic Neuropathies represent a type of disorder in which progressive, bilateral, and unpainful decrease of visual acuity appears [**2**,**4**,**5**,**8**]. Among the most important modifications is the affectation of the chromatic sense, described by most authors, which appears early and consists in the fact that patients notice that certain colors, particularly red, are less bright [**2**,**8**]; additionally, the visual field exam emphasizes the presence of a scotoma, initially nasal located, which increases in dimensions, surrounding the fixation point and the preservation of the peripheral visual field. Rarely, have other visual field defects been observed (paracentral scotomas) [**2**]; direct ophthalmoscopy might reveal no modifications initially. However, later on, edema, hyperemia, and even disc haemorrhage could be observed. When the disorder progressed, the following could be seen: optical disc pallor, the atrophy of the interpapillomacular region and in the final phases the lesions expanded, leading to total optical atrophy [**1**-**3**,**6**-**8**]. Toxic optic neuropathies can be triggered or intensified by a nutritional deficit of vitamin B1 (Thiamine), B2 (Riboflavin), B3 (Niacin), B6, B12 (Cobalamin) and Folic acid. Various toxins can also trigger or intensify the disorder, such as drugs, metals, organic solvents, methanol, carbon dioxide, ethanol, and nicotine [**3**].

The pathological mechanism of the alcohol-tobacco intoxication is unknown. It is presumed that the hydrocyanic acid and the free radicals in tobacco deteriorate the mitochondrial respiratory chain of the DNA, thus modifications of the mitochondrial morphology appear, which lead to demyelination [**2**,**3**].

In most cases, the primary lesion is not located in the optic nerve, but it is possible for it to be located at the level of the retina, chiasm, or even the optic tracts [**1**-**3**,**8**].

The therapy consists initially of the removal of the causative agent, after which the patient must stop smoking and/ or drinking alcohol, as well as be administered Vitamin B12 and folates, etc. This case showed that an early treatment and diet could reestablish the function of the optic nerve with the complete recovery of the visual function, without any complications [**2**,**7**,**8**].

## Case presentation

We presented the case of a 34-year-old non-smoker patient, with a sudden and unpainful decrease in bilateral visual acuity, without any other symptoms, early in the morning. The patient had consumed alcohol and 20 cigarettes for four hours.

The examination of the anterior segment and the measurement of the intraocular pressure did not reveal any modifications. The chromatic sense was also positive, while direct ophthalmoscopy showed bilateral nasal optic disc pallor.

At the moment of hospitalization, visual acuity was 0.1 for both eyes, without any correction and 1 with correction (-5 Dsf). Visual acuity improved during hospitalization, having reached 1 cc-1Dsf and at the moment of discharge it was 1 without correction.

The neurological examination was performed to exclude the possibility of some of the manifestations of nutritional deficiencies, the neurological consequences of pernicious anemia, or the toxicity of other substances and to determine whether other examinations were needed, such as the analysis of the cerebrospinal fluid. The neurological examination did not reveal any significant modifications, with the exception of a neurotic tremor. Vitamin B1 and B6 administration was recommended, as well as the examination of the visually evoked potential.

Because the exact cause could not be indicated from the medical history of the patient and to exclude other compressive or ischemic etiologies, the following procedures were performed: native cranial computer tomograph, craniocerebral and orbital MRI. The results did not show any pathological modifications.

The laboratory tests showed an increase of hepatic enzymes, glycemia, erythrocytes, hemoglobin, and hematocrit. The results indicated secondary polycythemia with the recommendation to repeat the hematological exam in six weeks.

The biochemical exam to detect high-risk drugs in urine was negative.

## Evolution

During hospitalization, the patient had a favorable evolution, with the complete recovery of his visual function, after having been treated with: antibiotics, steroidal anti-inflammatories, vitamins (B1 and B6) and Gingko Biloba.

## Discussions 

Tobacco-Alcohol Toxic Optic Neuropathy has a variable prognosis that depends on the level of exposure to the toxin, removing the toxin as quickly as possible and the visual acuity at the time of diagnosis. The patients without or with insignificant modifications observed via direct ophthalmoscopy have a better recovery than patients with ocular atrophy at the moment of diagnosis, as well as the patients with nutritional deficiencies. This is the case of the patient mentioned earlier, who presented in our medical service at the debut of his symptomatology, and thus his visual recovery was fast. In some cases, injections with Hydroxocobalamin had success in treating the disorder, even when the patients continued smoking [**2**,**8**].

After 4-6 weeks, an examination that includes testing the visual acuity, chromatic sense, direct ophthalmoscopy, the pupillary reaction, and the aspect of the optic papilla is necessary. Depending on the recovery, examinations are required after six months to one year [**8**].
